# NR1 Splicing Variant NR1a in Cerebellar Granule Neurons Constitutes a Better Motor Learning in the Mouse

**DOI:** 10.1007/s12311-023-01614-5

**Published:** 2023-10-25

**Authors:** Ting Tan, Linyan Jiang, Zhengxiao He, Xuejiao Ding, Xiaoli Xiong, Mingxi Tang, Yuan Chen, Yaping Tang

**Affiliations:** 1 Neurobiology Research Center, School of Medicine, Shenzhen Campus of Sun Yat-Sen University, Shenzhen, 518107 China; 2 Guangzhou Women and Children’s Medical Center, Guangzhou Institute of Pediatrics, Guangzhou Medical University, Guangzhou, 510623 China; 3 Department of Pathology, Affiliated Hospital of Southwest Medical University, Luzhou, 646000 Sichuan China

**Keywords:** Cerebellum, Granule cell, NR1a, Motor learning

## Abstract

As an excitatory neuron in the cerebellum, the granule cells play a crucial role in motor learning. The assembly of NMDAR in these neurons varies in developmental stages, while the significance of this variety is still not clear. In this study, we found that motor training could specially upregulate the expression level of NR1a, a splicing form of NR1 subunit. Interestingly, overexpression of this splicing variant in a cerebellar granule cell-specific manner dramatically elevated the NMDAR binding activity. Furthermore, the NR1a transgenic mice did not only show an enhanced motor learning, but also exhibit a higher efficacy for motor training in motor learning. Our results suggested that as a “junior” receptor, NR1a facilitates NMDAR activity as well as motor skill learning.

## Introduction

Motor skill learning is the improvement in speed, accuracy, or consistency of movement with training [1], and is characterized by slow and lifelong development that does not require conscious recall [2–4]. The cerebellum plays a crucial role in this process [5, 6]. Corresponding cerebellar regions are activated during different stages of motor learning. For example, high activation of the motor learning process occurs in the posterior lobes of the cerebellar hemispheres [7, 8]. When individuals perform a learned task as fast as they can, the activation increases in the right dentate nucleus and the right posterior hemispheres [9, 10]. Moreover, the capacity of the cerebellum to memorize motor skills is distinct from its ability to organize or coordinate motor activities [4, 11].

Cerebellar granule cells are the most numerous neuron types in the vertebrate brain [12]. They receive sensorimotor information via mossy fibers and then projection to Purkinje cells [13–15]. Many forms of motor learning substrates depend on this projection [16–19]. Moreover, injection of drugs inducing cerebellar granule cell apoptosis results in impaired motor learning in mice [20]. The importance of granule cell in motor learning has been confirmed by these studies. However, the mechanism of granule cell involvement in motor regulation is not fully understood.

Accumulating evidences indicate that N-methyl-D-aspartate receptor (NMDAR) complex plays a role in motor skill and motor learning [21–24]. Accurate modulation of NMDAR is necessary for effective motor skills, and dysfunction of these receptors may lead to impaired motor skills [25–27]. NMDAR complexes are assembled by a diverse array of 4 distinct function subunits (GluN1, GluN2A-D, and GluN3A-B) [28–30]. GluN1 subunit is necessary for each glutamate receptor heterodimer to anchor GluN2 or GluN3 subunits, comprising a functional ionic channel. GluN1 contains distinct domains, which can bind different proteins to cellular plasma membrane [31] and activate downstream signaling pathways [32]. Our previous work on mice hippocampus demonstrated that recombinant NMDAR with NR2b overexpression enhanced synaptic plasticity as well as hippocampal-dependent learning [33]. Additionally, recombinant NMDAR with NR2b overexpression in cerebellar granule cell-specific mice enhanced age-dependent and motor learning-specific function [34]. Therefore, it is important to determine whether other different recombinant NMDARs alter cerebellar learning.

## Materials and Methods

### Immunofluorescence

Mice were fully anesthetized and transcardially perfused with 0.9% saline followed by 4% paraformaldehyde (PFA) in phosphate buffer. Brains were fixed overnight in 4% PFA. Brain sections were initially pre-incubated in phosphate buffer with 3‰ Triton X-100 and 5% bovine serum albumin. Then, sections were incubated in c-fos (Abcam, 1:500) antibody overnight at 4 °C and incubated in secondary antibody (Abcam, 1:1000) for 2 h at room temperature. Finally, all sections were sealed with antifade mounting medium with DAPI (Sigma-Aldrich).

### Laser Microdissection (LMD)

The brain was sequentially sliced into 12 μm sections. Sections were washed twice in 80% ethanol to remove the OCT and stained in cresyl violet (MedChemExpress) solution. Then sections were immersed in 80%, 95%, 100% ethanol and xylene respectively and kept no RNase contamination during all operations. Sections were microdissected using the LMD6500 system (Leica microsystems). Equal amounts of enriched granule cells were lasered in each session. Samples were collected in lysis solution and stored at − 80 °C.

### Quantitative Real-Time Reverse Transcriptase-PCR

Total RNA extraction was performed using TRIzol reagent (Invitrogen). RNA concentration and quality were measured using a spectrophotometer, and only samples with 260/280 and 260/230 ratios > 1.8 were accepted. cDNA was synthesized starting from 1 μg of the extracted RNA using cDNA Synthesis Kit for RT-qPCR (Takara), following the manufacturer’s indications. The resulting product was diluted 1:10, and 1 μL was used as the template for RT-qPCR. qPCR analysis was completed using SYBR Green Mix (Takara) on a CFX96 thermal cycler (BioRad). The primers were as follows: NR1a (forward cgtgagtccaaggcagagaa, reverse tcgtcctcgcttgcagaaa); NR1b (forward agcgtgagtccaagagtaa, reverse gtcgtcctcgcttgcagaa); c-fos (forward atggtgaagaccgtgtcagg, reverse tcagctccctcctccgattc).

### In Situ* Hybridization*

We used in situ hybridization with a ^35^S-labeled oligo probe that could detect the NR1a and NR1b expression pattern. The procedures were described in our previous publication [33]. The probe sequences were referenced from publications [35, 36].

### Generation of Cerebellar Granule Cell-Specific NR1a Transgenic Mice

The procedures were similar to our previous publication [34]. GABA-a6-tTA construct consisted of a cerebellar granule cell-specific promoter GABA-a6, an IRES element, tTA, and SV-40 poly-A signal. The tetO-NR1a construct consisted of the tetO mini promoter, the artificial intron, mouse NR1a cDNA, and the SV-40 poly-A. The transgenic cassettes were released by enzyme and purified away from plasmid sequence. The transgenic founders were produced by pronuclear injection of the linearized DNA into C57B/6 inbred zygotes as described. The inbred founders were crossed into C57B/6 to produce F1 generation. The F2 offsprings derived from intercross between GABA-a6-tTA and tetO-NR1a transgenic mice were used for various analyses. The genotypes after the F1 generation were determined by PCR analysis of tail DNA with primers respectively for tTA transgene and NR1a (SV-40) transgene.

### Western Blot

Total proteins were extracted using RIPA lysis buffer (Beyotime) and quantified using Pierce BCA Protein Assay Kit (Thermo Fisher). Proteins were separated by SDS-PAGE and transferred onto PVDF membrane. The membrane was blocked in defatted milk powder and incubated at 4 ℃ overnight in NR1 primary antibody (Cell Signaling Technology, 1:1000). Membranes were washed in PBST 5 min for 3 times and then incubated in secondary antibody (Bioss, 1:5000) for 1 h at room temperature. ECL chromogenic substrate (Millipore) was added for development and was imaged using a gel imaging system.

### Quantitative Autoradiography

[^3^H] MK-801 binding was performed as previously described [37, 38]. Briefly, cerebellums were homogenized and centrifuged. The pellets were suspended and incubated with EDTA-Tris solution after washing with ultra-pure water. Protein concentration was determined by the BCA protein kit. Finally, proteins were incubated with equal amount of [3H] MK-801 assay buffer. 10 μm thick brain sections were pre-incubated in Tris-HCl buffer containing CaCl2 for 10 min at 4 ℃ and then incubated in [3H]MK 801 buffer for 60 min at room temperature. Then, sections were rinsed with water and glutaraldehyde. The dried sections were exposed to Hyperfilm. After exposure, the films were developed, fixed, and dried.

### Motor Learning in Rotarod Task and MK-801 Injection

3-month-old mice was used to test cell activation after fixed-speed rotarod training (35 rpm, once a day for 3 consecutive days). The rotarod trials were performed at 15 min after MK-801 (2 mg/kg, Sigma-Aldrich) intraperitoneal injection in another group which finished fixed-speed training for consecutive 3 days.

Fixed-speed training was performed in 3-week-old mice (15 rpm) and 3-month-old mice (35 rpm) for consecutive 3 days to detect the expression of NR1a/b in mice of different ages. Slower rotarod speed was used for weaning 3-week-old mice to ensure that they could complete the test. Cerebellum tissues were retained at different time points after trials on the 4th day.

For transgenic mice, 3 fixed-speed rotarod trials (35 rpm) were used per day for 3 consecutive days. The test trials are performed on the 4th day, and the average time on the 4th day was calculated. The self-training and accelerated-speed rotarod procedures for transgenic mice were similar to our previous publication [34].

## Results

### Cerebellar Cortex Granule Cells Are Activated Rapidly by Rotarod Training

**Fig. 1:**
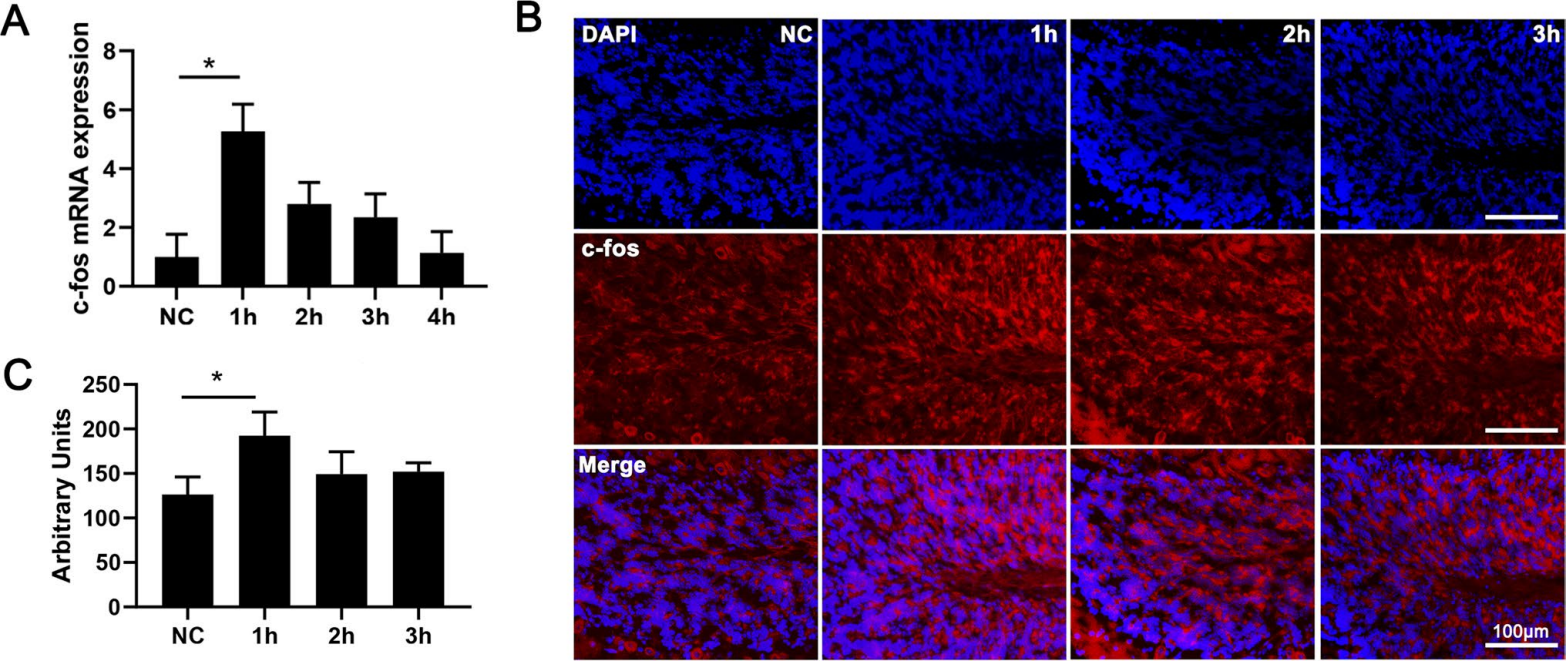
Activated cerebellar granule cells in rotarod-trained mice. **A** Expression of c-fos mRNA in mice cerebellum at negative control (NC) 1h/2h/3h/4h (*n* = 3) after rotarod training. **B** c-fos immunofluorescence of negative control mice (untrained mice) and rotarod-trained mice in cerebellum cortex. Scale bar: 100 μm. **C** Quantitative statistics of fluorescence density of **c-fos**. NC, /1h /2h /3h (*n* = 6). Data are expressed as mean ± SEM, *P < 0.05

The vertebrate cerebellum is involved in multiple aspects of motor coordination [39], and the first stage of refining motor output occurs in the granule cell layer [40]. c-fos gene encodes the transcription factor that regulates the activity of effector genes at the early stage after stimulus [41]. Therefore, c-fos is used to label activated granule cells when verifying the association between motor activity and cerebellar granule cells [42]. In our study, c-fos mRNA expression was detected at 1 h, 2 h, 3 h, and 4 h separately after rotarod training. The result showed that c-fos mRNA was significantly upregulated and reached a peak at 1 h after training and then began to decline and remained at NC group level 4 h later (Fig. 1A). c-fos protein expression trend was consistent with mRNA expression level which reached the peak at 1 h and then gradually decreased to NC level (Fig. 1B, C).

### Cerebellar Cortex Granule Cells Are Activated Through NMDAR

**Fig. 2:**
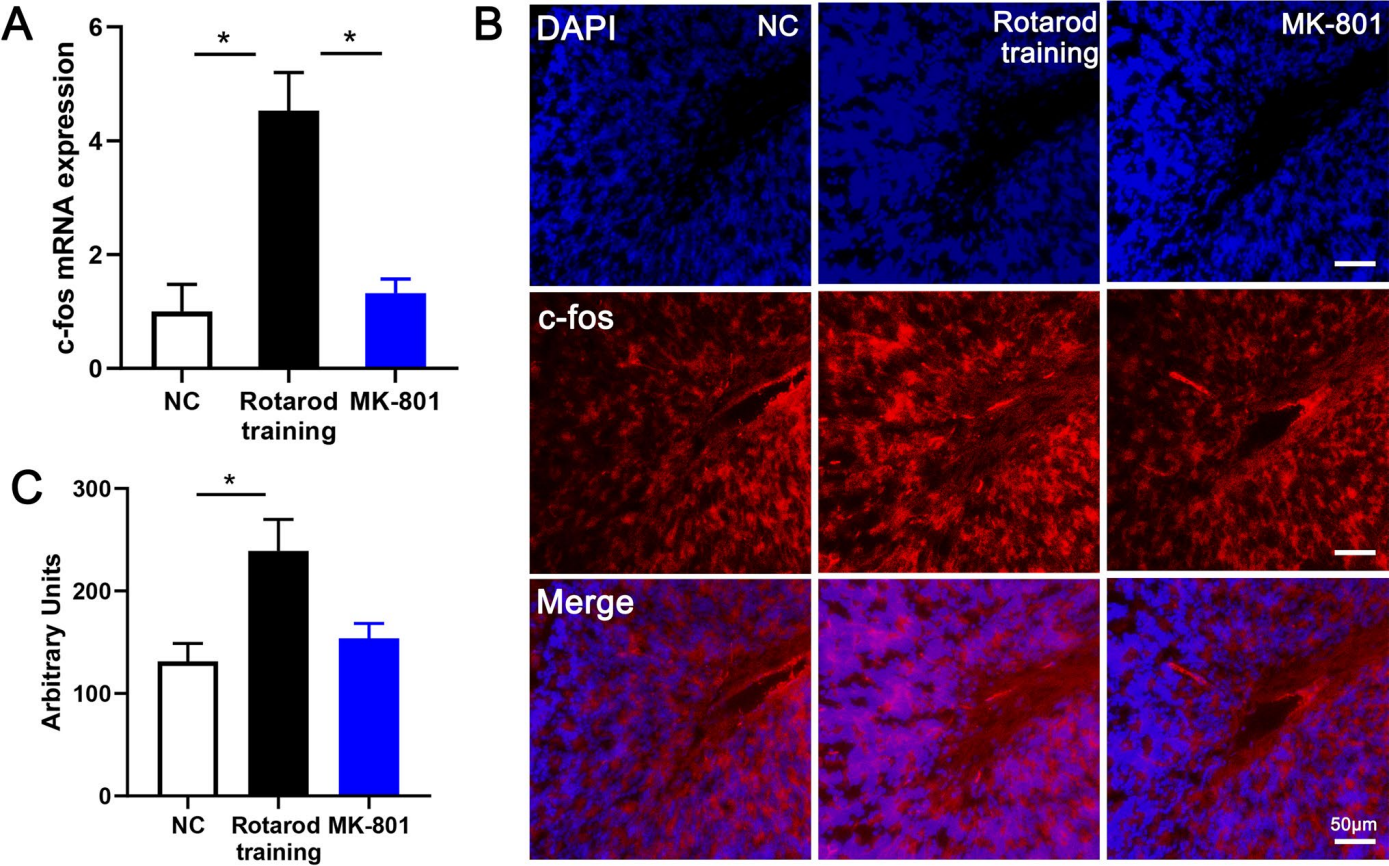
NMDAR antagonist inhibits activation of granule cell. **A** Expression of c-fos mRNA in mice cerebellum of three groups: NC, negative control without training; rotarod training, collecting cerebellum tissue at 1 h after training; MK-801, injecting MK-801 before training and collecting cerebellum tissue at 1 h after training. **B** c-fos immunofluorescence of NC/rotarod training/MK-801 mice in cerebellum cortex. Scale bar: 50 μm. **C** Quantitative statistics of fluorescence density of **B**. NC (*n* = 5)/rotarod training (*n* = 5)/MK-801 (*n* = 5). Data are expressed as mean ± SEM, *P < 0.05

Cerebellar granule cells receive glutamatergic projections from mossy fibers, and activation of NMDAR plays an important role in the physiological function of these synapses [43]. Moreover, NMDAR has been reported to involve in c-fos activation in some animal models. [44, 45]. To verify whether rotarod training-induced c-fos activation was associated with NMDAR, MK-801 (an NMDA antagonist) was injected before rotarod training. c-fos mRNA was significantly upregulated 4.2 times after training to NC, which could be inhibited by MK-801 (Fig. 2A). c-fos protein expression change was consistent with mRNA level (Fig. 2B, C).

In conclusion, rotarod training can rapidly activate cerebellar granulosa cells through NMDAR.

**Fig. 3:**
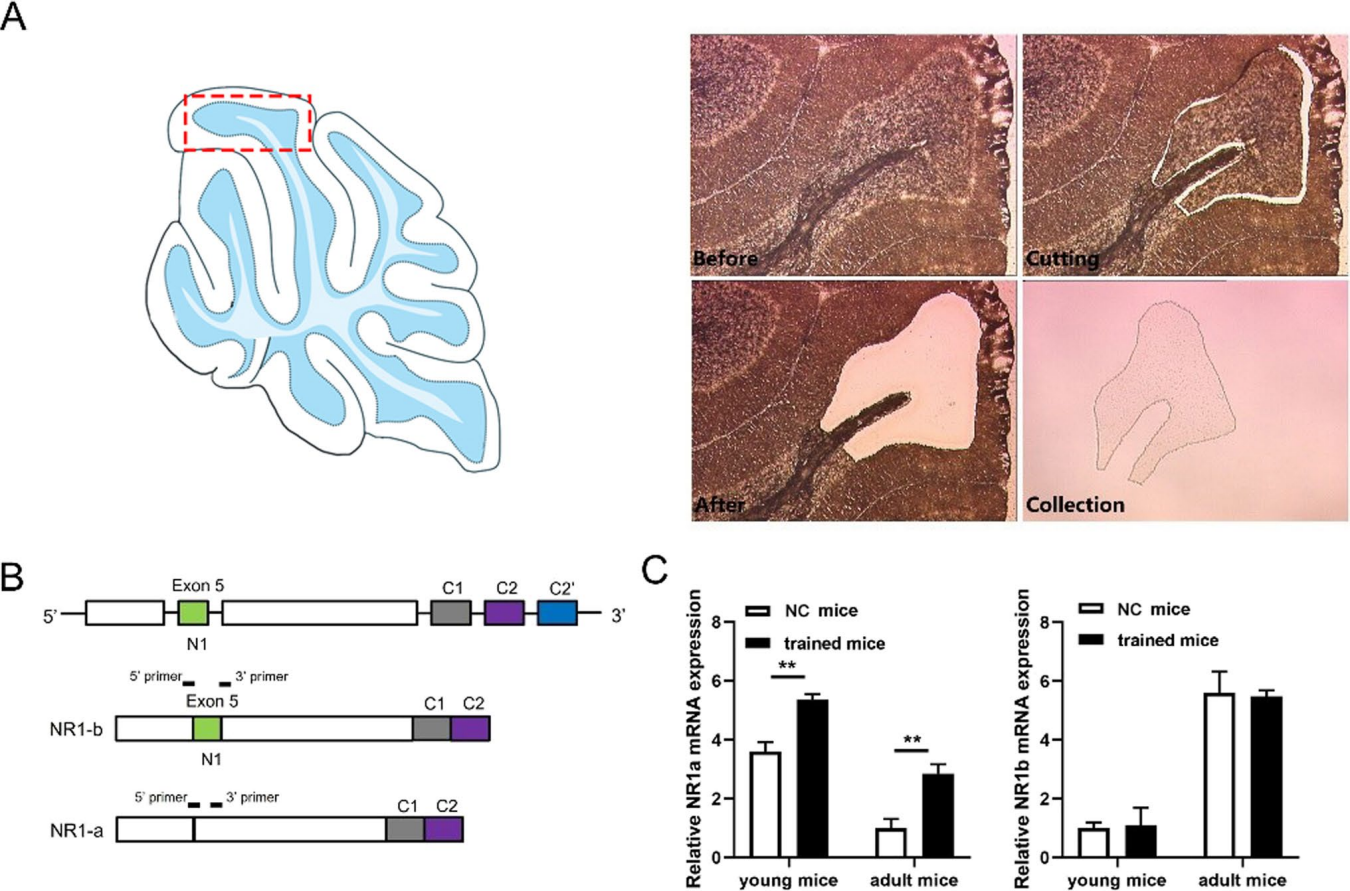
NR1a/b mRNA expression in rotarod-trained mice. **A** Laser capture microdissection diagram of cerebellar cortex granule cell area. **B** Top: partial diagram of the NR1 gene. Exon 5 encodes the N1 terminal domain of the NR1. Middle: schematic diagram of the NR1b (with exon5) splice variants. Bottom: schematic diagram of the NR1a (without exon5) splice variants. The position of primers is shown with bold lines on top of each splice variant. **C** mRNA expression of NR1a/b in the cerebellum. NC young mice (*n* = 4)/NC adult mice (*n* = 4)/trained young mice (*n* = 4)/trained adult mice (*n* = 4). Data are expressed as mean ± SEM, **P < 0.001; young mice, 3 weeks; adult mice, 3 months

The N1 cassette (exon 5) determines NMDAR properties [46], and high granule cellular activity changes the NR1a/NR1b ratio in vitro [47]. Here, we examined the expression of NR1a (without the N1 cassette) and NR1b (with N1 the cassette) (Fig. 3B) in NC and trained mice. Activated cerebellar granule cells were collected precisely through laser capture microdissection by using fast and efficient cresyl violet staining (Fig. 3A). We cut cerebellar lobe IV/V for RNA extraction according to research which showed that lobe IV/V is involved in the regulation of motor coordination in mice [48].

The result indicated that NR1a and NR1b mRNA presented opposite trend during the development of mice. NR1a mRNA is predominated in young mice and replaced by NR1b in adulthood. In addition, the expression of NR1a mRNA was substantially upregulated in the trained group compared with the NC group (Fig. 3C). Study has shown that young mice have better performance in motor skills and motor learning [49], which is consistent with NR1a/NR1b pattern change. These indicate that NR1α may be involved in the activation of cerebellar granule cells by rotarod training.

### Generation of Cerebellar Granule Cell-Specific NR1a Transgenic Mice

**Fig. 4:**
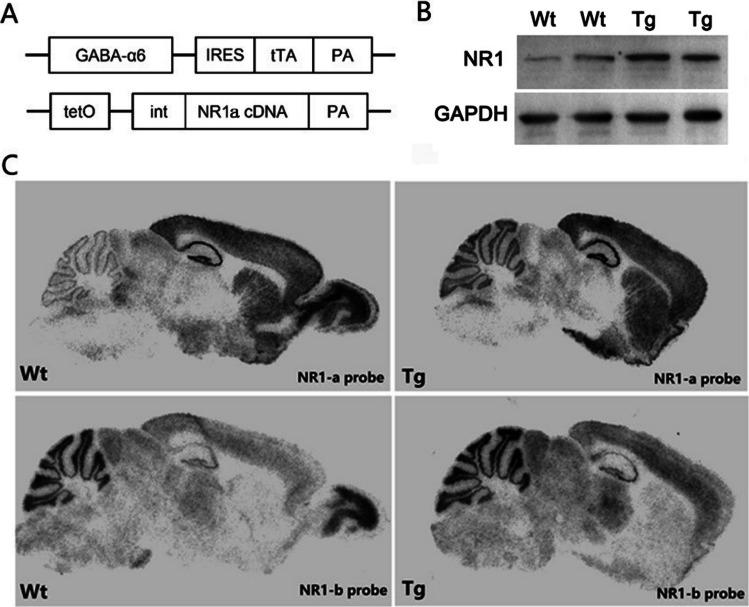
Inducible cerebellar granule cell-specific NR1a transgenic mice. **A** Expression vectors for GABA-α6-tTA (upper panel) and tetO-NR1a (low panel). pA, poly-A signal; int, artificial intron. **B** NR1 protein expression in transgene mice cerebellum. **C** Distribution of the NR1a/b in transgene mice cerebellum

According to our previous study, we generated inducible cerebellar granule cell-specific NR1a transgenic mice. Firstly, we generated two single transgenic mouse strains (Fig. 4A), with GABA-a6 as the granule cell-specific promoter used to drive tTA. We confirmed the specificity and efficacy of this promoter in our previous work [34]. Secondly, GABA-a6-tTA/tetO-NR1a double-transgenic mice were produced. Since there is no antibody that can accurately distinguish between NR1a and NR1b proteins, Western blotting was used to confirm the overexpression of the NR1 protein in the cerebellum of transgenic mice (Fig. 4B). In situ hybridization showed that NR1a was observed in cerebellar cortex of Tg mice, but not in Wt mice; same expression of NR1b is observed in Tg and Wt mice (Fig. 4C). This result confirmed that NR1a is overexpressed in granule cells of transgenic mice specifically. This mouse model was used in the following experiments to verify the effect of NR1a overexpressed recombinant NDMAR on motor learning.

### *Elevated [*^*3*^*H]-MK801 Binding Activity in Transgenic Cerebellum*

**Fig. 5:**
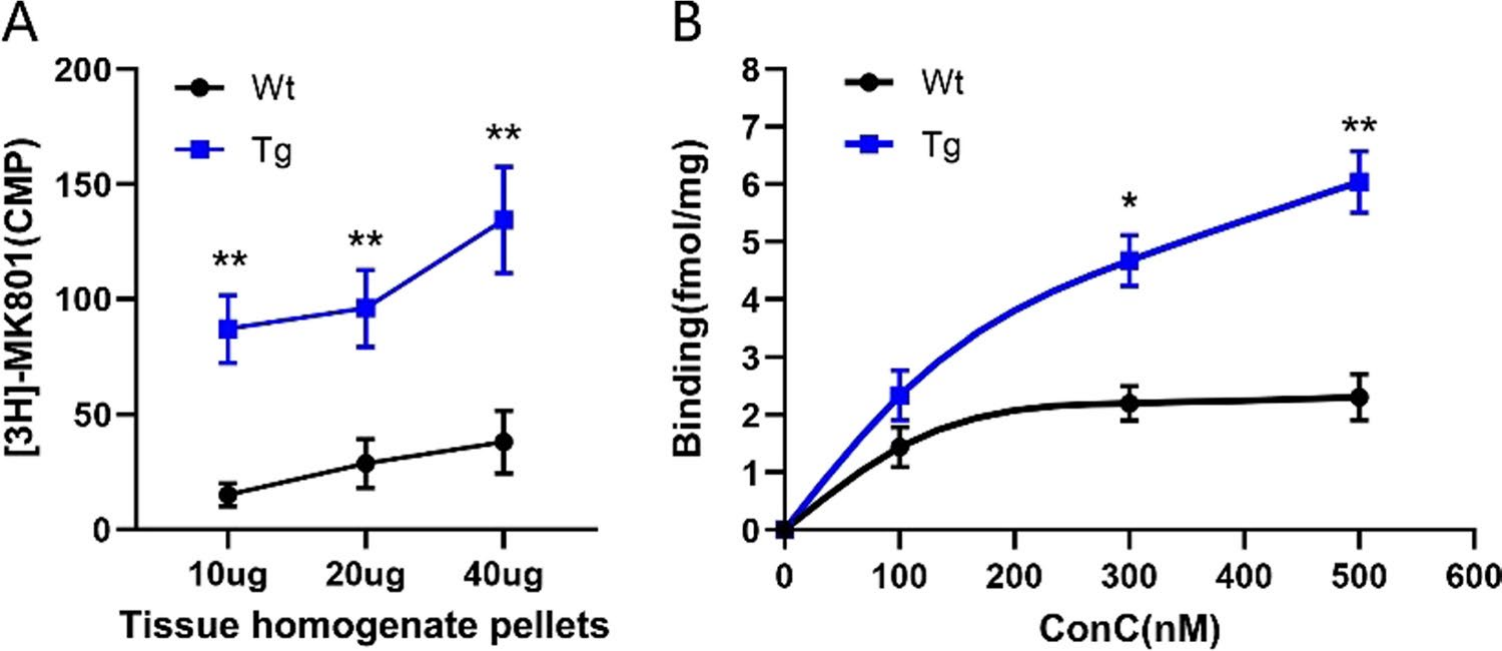
Binding of [.^3^H]-MK801 to NMDAR in transgenic mice. **A** Tissue dilution curves of [3H]-MK801 binding to NMDAR in Wt and Tg mice cerebellar homogenate. **B** Binding curves of [3H]-MK801 binding to NMDAR in Wt and Tg mice cerebellar slices. Wt (*n* = 4) and Tg (*n* = 4). The higher binding ratio in Tg cerebellum is verified by two different assayed methods. Data are expressed as mean ± SEM; **P < 0.01, *P < 0.05

MK801 is an NMDAR noncompetitive antagonist [50] that specifically binds to the channel blocking site of the NMDAR [51]. Based on this binding feature, [^3^H]-MK801 was used to measure the amount and activity of NMDAR. Incubation of the cerebellar homogenates with [^3^H]-MK80 showed that binding to [^3^H]-MK801 in transgenic mice was substantially higher than that in wild-type mice (Fig. 5A). After incubation of the brain slices, the binding of NMDAR reached saturation when the concentration of [^3^H]-MK801 reached 200 nM in the wild-type group. However, the binding in transgenic mice increased persistently with increasing in [^3^H]-MK801 concentration (Fig. 5B). These results indicated that the amount and activity of NMDAR are significantly elevated in transgenic cerebellum. These data further verified the success of these transgenic mice and ensured the reliability of the results of subsequent behavioral tests.

### Enhanced Motor Controlling Ability and Motor Learning in Transgenic Mice with Overexpressed NR1a

**Fig. 6:**
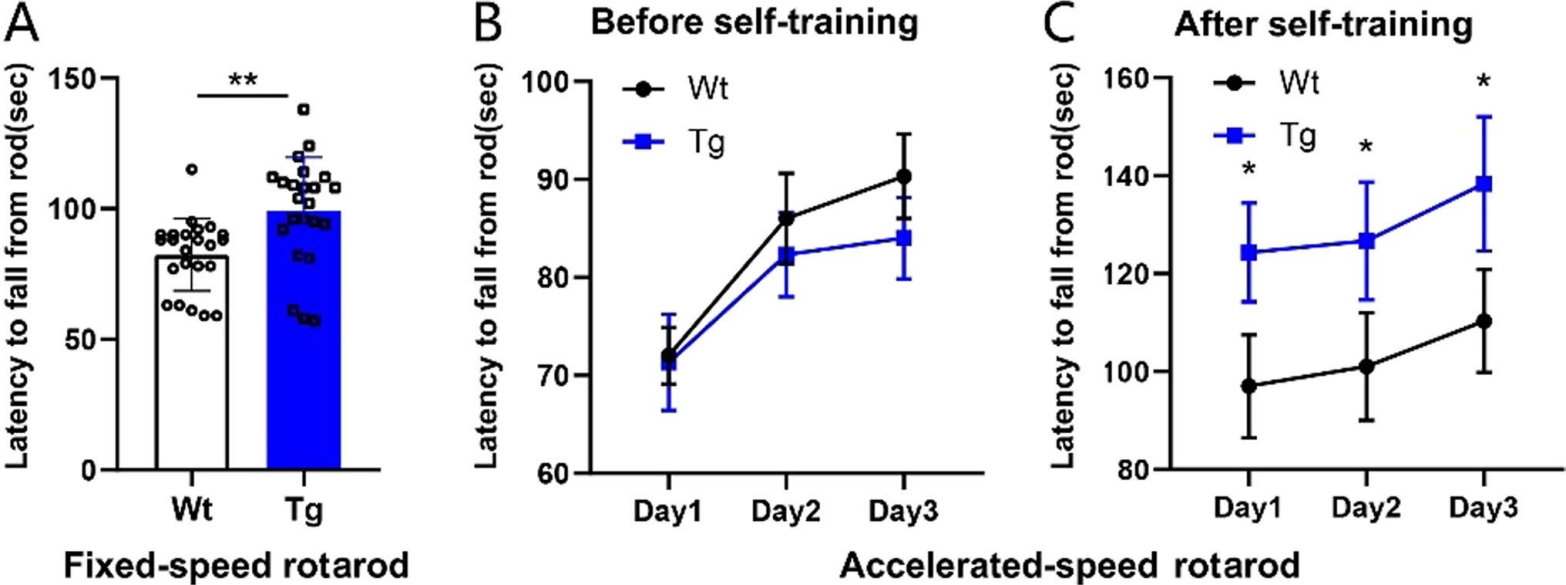
Effects of the NR1a transgene on motor learning. **A** Latency to fall of mice in a fixed-speed rotarod. Wt (*n* = 23)/Tg (*n* = 23). **B** Motor learning in an accelerated-speed rotarod test. Two different sets of mice were respectively used for fixed- and accelerated-speed rotarod tests. Data are expressed as mean ± SEM; **P < 0.01, *P < 0.05

Transgenic mice spent more time on the rod than wild-type mice in the fixed-speed rotarod test (Fig. 6A). This data suggests that the involvement of cerebellar granule cells in the regulation of motor skill control occurs through NR1a. Then, we used an accelerated rotarod training test to examine whether motor skill learning changed in transgenic mice. The time spent on the rod gradually increases each day in both Wt and Tg groups without self-training. However, there was no significant difference between the two groups at each day. In contrast, the time spent on the rod by the transgenic mice was considerably higher than that spent by wild-type mice after self-training (Fig. 6B) per test day. This indicates that NR1a might be responsible for the enhancement of motor skill learning.

## Discussion

We demonstrated that transgenic mice with overexpression of NR1a in the cerebellar granule cells undergoing rotarod training could enhance motor learning. Granule cells are the major dopaminergic excitatory cells in the cerebellar cortex and originate in the upper rhombic lip [52, 53]. Excitatory granule cells make up the cerebellar granular layer. Interaction of these cells with inhibitory Golgi cells can help determine network responses to external stimuli [54]. Hence, a functional change in granule cells may directly affect information integration within the cerebellar computational network and subsequently affect motor learning. To our knowledge, this is the first study to demonstrate that the NR1a subunit of NMDAR in granule cells constitutes a molecular basis for the involvement of the cerebellum to facilitate the development of better motor learning. Meanwhile, mouse models with cortex CA1 hippocampus/striatum-specific removal of the NR1 subunit have provided considerable information on the role of somatosensory pattern development and synaptic plasticity in spatial memory [55–57]. These results indicate that motor skill learning is coordinated by multiple brain regions. Therefore, our cerebellum granule cell-specific NR1a overexpression mice could serve as a valuable tool for studying other cerebellar functions.

This is the first study to confirm the importance of the NR1a subunit in motor skill learning related to rotarod performance. Without the intervention of environmental factors, recombinant NMDAR-overexpressing NR1a transgenic mice could obtain elevated motor skills, whereas our previous NR2b recombinant mice did not show such a change. This result suggests that NR1a plays a critical role in the development of motor skills. NR1 is often considered indispensable for functional NMDAR assemblies [35], and alternative splicing of NR1 subunit mRNA has substantial effects on the NMDA receptor properties [58]. In addition, exon 5 of NR1 is an N-terminal splicing cassette whose expression is strongly regulated throughout development. The NR1 subunit transitions from the NR1a form (without exon 5) in the embryonic stage to the NR1b (with exon 5) form in adulthood [46, 59]. This age-dependent expression pattern may be induced by functional differences and neuron activity. For example, Mary et al. demonstrated that high levels of granule cell activity with special culture systems inhibit the expression of NR1b in vitro [47]. Moreover, NR1a and NR1b show largely varying affinity to agonists, potentiation to zinc and magnesium ions, current amplitudes, sensitivity to proton inhibition, and response to polyamines and protein kinase C [36, 46, 60–63]. These differences may be the molecular basis for functional diversity.

Long-term synaptic plasticity serves as a base for learning and memory. Fast motor skill learning requires acceleration of rotarod tasks and modulating synaptic efficacy through long-term potentiation and long-term depression in rodents [64, 65]. Martijn et al. demonstrated that NMDARs are necessary for LTP and LTD induction of parallel fiber-Purkinje cell (PF-PC) synapses for cerebellar motor learning by specifically deleting NR1 [16]. In our previous study, we developed recombinant NMDAR NR2b transgenic mice and demonstrated that granule cells facilitate the development of synaptic use-dependent plasticity [34]. However, the change and potential implications of synaptic plasticity in NR1a transgenic mice warrant further study.

## Conclusion

Our results validated that expression of NR1a in the cerebellar granule cells may constitute a molecular basis for NMDAR activity and motor skill learning.

## Data Availability

The datasets in this study are available from the corresponding author on request.
